# Health, well-being, and measuring the burden of disease

**DOI:** 10.1186/1478-7954-10-13

**Published:** 2012-08-01

**Authors:** Daniel M Hausman

**Affiliations:** 1 Department of Philosophy, University of Wisconsin-Madison, Helen C White Hall, 600 N. Park Street, Madison, WI 53706, USA

**Keywords:** Burden of disease, Well-being, Health measurement

## Abstract

This essay asks whether the global burden of diseases, injuries, and risk factors (GBD) should be measured in terms of their consequences for health, as maintained by most of those who are attempting to measure the GBD, or in terms of their consequences for well-being, as argued by John Broome. It answers that the burden of disease should be understood in terms of the consequences of disease for health, and it defends the wider efforts to measure health by those who are in other ways skeptical of the project of measuring the GBD.

## 

An important project spearheaded by some of the World Health Organization (WHO) staff in the 1990s was to measure the global “burden of disease” – that is, the contributions of diseases, injuries, and risk factors such as tobacco smoking to ill health. This international effort continues at several institutions and especially in the work of the Institute for Health Metrics and Evaluation (IHME). At the risk of implying more cohesiveness and unity than is in fact the case, I shall refer to these researchers as “the GBD team.” To measure the global burden of disease, the GBD team has attempted to generate summary measures of overall population health and to measure the health effects of diseases by means of these summary measures. The GBD team hopes that these summary measures will serve other purposes, such as identifying locations where health is particularly bad, assisting research, and guiding the allocation of health-related resources ([1]), but this essay is concerned mainly with the attempt to measure the global burden of diseases, injuries, and risk factors.

In particular, this essay asks whether the global burden of diseases, injuries, and risk factors should be understood in terms of their consequences for health, as maintained by the GBD team, or in terms of their consequences for well-being, as argued by John Broome [2]. I answer that the burden of disease should be understood in terms of the consequences of disease for health, and I defend the wider efforts to measure health by many others who are in other ways skeptical of detailed features of the GBD team’s projects.

The view of the burden of disease shared by the GBD team and by most others who have attempted to measure health starts with conceptualizing a person’s health over a time period in terms of the sequence of their health states. People’s health states are defined in turn by their functional deficiencies (such as cognitive problems, limitations to mobility or agility, sensory deficiencies, or affective disorders), and by aspects of their subjective states, such as pain and depression. Diseases, injuries, and risk factors, like health interventions, change the distribution of health states within a population. This framework abstracts as far as possible from the debates concerning the concept of health. It is compatible with the so-called “biostatistical view” defended by Christopher Boorse ([3-6]) and the related views defended by Jerome Wakefield ([7,8]). But is also compatible with many more evaluative views of health. (For general discussions of the concept of health see [9] and [10].) The burden of disease understood concretely is the contrast between the distribution of health states due to some disease or risk factor and a state of complete health. Without a scalar measure of the change in health states, there is however no unambiguous way to compare the burdens of different causes of ill health. So what is called the burden of disease is some scalar measure of the departure from full health due to disease.

The GBD team, unlike those who have generated health measurement schemes such as the EQ-5D or the Health Utilities Index (HUI), has hoped literally to measure the quantity of health. For example, Mathers et al. [11], p. 324 write, “The health state valuations . . . represent quantifications of the overall health levels associated with different states.” Salomon et al. [12], p. 307 write, “[W]e consider a health state valuation to provide a scalar cardinal index of the overall level of health associated with a multidimensional health state.” (See also [13], p. 16, and [14], p. 431.) But it is impossible literally to measure the quantity of health, because the relation “healthier than” is incomplete. Health is multidimensional, and the different dimensions are not commensurate. The same is true of commodity bundles, and just as there is no way to say whether one bundle of commodities is larger than another (unless the first contains at least as much of each commodity as the second), there is no way to say whether one health state contains “more health” than another ([15]). Though there is no way to assign a measure to some putative quantity of health itself (just as there is no way to measure the size of a commodity bundle), people can *evaluate* health states (just as they can evaluate commodity bundles). By treating the values of health states as their measures – that is, by measuring health states by how good they are – health economists can calculate the values of distributions of health states. Health economists can then measure the effect of a disease on population health by subtracting the value of the distribution of health states that results from the disease from an estimate of the value of what the distribution would be without disease.

John Broome argues that this way of determining the global burden of disease is misconceived ([2]). He maintains that those concerned with the burden of disease should attempt to measure the effects of diseases on well-being rather than attempting to measure their health effects. Although he directs his criticisms to the GBD team, they apply broadly to efforts to measure health, both welfarist and “extra-welfarist” ([16]). Broome maintains that purported measurements of health are really defective measures of well-being, and his arguments imply that health cannot be measured at all. Broome’s position is implicitly as critical of those who attack the GBD team’s efforts, such as Alan Williams ([17]), as of the GBD team’s project.

Broome makes two arguments against measuring the burden of disease by its consequences for health. His first argument relies on the premise that the value of health is its contribution to well-being.

". . . the measure should measure health as a component of well-being . . . . it is to measure how good a person’s health is for the person, or how bad her ill-health. . . . That is to say, it aims to measure the contribution of health to well-being. ([2], p. 94)"

The argument then concludes that the value of health cannot be measured. Here is the argument: Health contributes to well-being both causally and constitutively. But the contribution that health makes to well-being is not separable from the contribution of other factors. Individuals in the same health state but different circumstances will often not be equally well off, and that difference cannot be factored into some common portion contributed by health coupled with the separate contribution of the circumstances. The contribution to well-being of a token health state of a particular kind (that is, the contribution to overall well-being of a specific person being in that health state at a specific place and time) differs depending on a person’s circumstances. A health state of any specific kind makes no uniform contribution to well-being.

"Obviously, the way in which a person’s well-being is affected by the various elements of her health depends a great deal on other features of her life. For example, asthma is less bad if you are well housed, mental handicap less bad in supportive communities, blindness less bad if you have access to the internet. ([2], p. 95)"

So there can be no measure of the well-being produced by a kind of health state.

In fact, Broome’s critique is much more general. Whether or not one focuses on well-being, the effects of health states that determine their values depend on the geographical, economic, technological, and cultural environment. In addition, the values of health states depend on the tastes, values, and objectives of individuals and on prevailing social values. Because health states have different evaluatively relevant consequences in different contexts, token health states will have different values, and health states of a given kind will have no uniform value at all. Since the value of a kind or type of health state is not defined, it cannot be measured. *There is no such thing as the value of a kind of health state*. So there is no way to measure the burden of disease by measuring the value of the distribution of the kinds of health states it causes. Health cannot be measured either by its quantity or its value. Indeed, it cannot be measured at all!

Although not stating his negative argument this broadly, Broome concludes that the burden of disease should be measured by the change in well-being it causes in the circumstances. If it is impossible to measure health, then one cannot measure the burden of disease by measuring health. If what matters is well-being (which leads to Broome’s second argument), then one should focus on the joint consequences for well-being of health states and nonhealth factors.

Broome’s second argument against measuring the burden of disease by its consequences for health is that “we should be concerned with all of well-being” ([2], p. 98) and hence with “the whole reduction in people’s well-being, which is caused by disease” ([2], p. 97) not with “only the part that consists in a reduction in people’s health” ([2], p. 93). I take the “we” here to include those who make health policy. The implications of this view extend far beyond efforts to carry out summary measures of population health. For example, Broome’s view implies that the National Institute for Health and Clinical Excellence should determine what treatments the National Health Service should provide by examining their effectiveness at promoting well-being, rather than by examining their effectiveness at promoting health.

Broome maintains that health policy must be guided by considerations of fairness as well as by concerns about well-being. Saving the life of a poor patient or a disabled patient may contribute less to aggregate well-being than saving the life of someone richer or not disabled, but that does not, in Broome’s view, justify treating the rich or nondisabled rather than the poor or disabled, because doing so would be unfair ([2], p. 99). Although Broome believes that fairness ultimately matters because of its contribution to the overall good ([2], p. 100), he maintains that it is useful to separate concerns about fairness from concerns about increasing well-being. In Broome’s view one should measure the burden of disease by the consequences for both well-being and fairness. His focus in this critique is, however, on well-being. So, Broome argues, the burden of disease should be quantified by the impact of disease on well-being, and the value of a token health state lies in its bearing on well-being. Broome writes, for example,

"Disease causes harms of a great many sorts, which are often not themselves specifically changes in health. For example, some diseases prevent their victims from working, and so deprive them of income and the other benefits that accompany work: companionship, self-esteem and so on."

"Indeed, the harms that *are* always treated as changes in health often consist in deprivation of goods other than health. . . ."

"So we ought not to be trying to measure the harm done by disease in terms of health only, but in terms of the whole of well-being. ([2], p. 98)"

Up until the last sentence, the quotation seems correct, indeed obviously so – provided that one does not forget that the direct effects of disease are virtually entirely effects on health and that the other harms that diseases cause are the indirect effects of the changes in health they cause. But the conclusion that those concerned with health policy should be measuring the harm done by disease “in terms of the whole of well-being” does not follow without additional premises.

Health matters to people in many ways. For example, certain health states damage or destroy people’s ability to manage their own lives. In doing so, they typically also diminish well-being, but one may reasonably question whether the value of a loss of autonomy is captured by the extent to which that loss of autonomy diminishes well-being. Other health states interfere with people’s abilities to pursue objectives that they value. The inability to pursue those objectives will not necessarily diminish people’s well-being, because the objectives people pursue are often irrelevant to their well-being and sometimes even detrimental to it. A young man who is too ill to join the army to help resist an enemy invasion may be better off for his illness, even if he is deeply committed to sharing in the effort at defense. And even when limitations on people’s ability to pursue their objectives diminish their well-being – as they typically do – the significance of the limitation need not coincide with the extent to which those limitations diminish well-being.

In practice, economists assign values to health states by measuring people’s preferences among them. These preferences do not always coincide with people’s judgments of what would be best for themselves nor with what would in fact be best for them ([18], chapters 7 and 8). In fact, the problematic actual methods of assigning values to the consequences of disease by measuring preferences neither focus exclusively on the consequences of disease for well-being nor on the consequences of disease specifically for health.

It might be argued that even though the bearing of health states on well-being does not exhaust the ways in which health states matter to individuals, only their bearing on well-being should influence social policy. *If* the sole ultimate goal of social policy were to enhance welfare (within the limits of fairness), then it would seem that the relevant burden of disease consists of the consequences of disease for well-being.

Many would reject the view of social policy as aimed ultimately at promoting welfare. Libertarians argue that the ultimate aim of social policy is to enhance individual freedom, and that people’s well-being is their own responsibility, not at all a social concern. Most liberals concede that one aim of social policy is to promote welfare, but insist that policies have other independent aims, including protecting freedom and expanding opportunities. Since health influences opportunity and people’s ability to make use of their freedom, liberals should deny that the value of health is exhausted by its bearing on well-being.

Even if one held that the ultimate aim of social policy is to promote well-being within the constraints of fairness, it does not follow that the burden of disease should be measured by the impact of disease on well-being. To enhance well-being within the constraints of fairness, policymakers need information about the consequences of alternative policies. Information about the magnitudes of problems and the consequences of policies may be useful even if it does not specify how those consequences bear on individual well-being. Simply knowing how many people die every year of malaria and at what ages tells policymakers that malaria is a very serious problem even though this information makes no explicit claims about well-being. From the premise that policymakers need to know the consequences of alternative health policies for well-being, it does not follow that health should be measured in terms of well-being. All that follows is that policymakers be able to draw inferences concerning well-being from measures of health.

Furthermore, regardless of the ultimate aims, social policy has many interim aims, and with respect to the interim aims, measures of health states in terms of their impact on overall well-being or fairness may be inferior to other health state measures. Suppose, for example, that a government is attempting to diminish poverty and that it wants information about the health of members of social groups both to diagnose how serious the poverty of different groups may be (since poverty affects health) and because it is considering undertaking public health measures as a way of addressing poverty (since health affects poverty). Information concerning the well-being of social groups is not what is needed, nor is information about the effects on overall well-being of the contemplated public health initiatives. In measuring health, health analysts are generating data that will be used for many purposes, and Broome has provided no argument for the claim that information concerning the impact of health states for well-being will serve these purposes better than other sorts of information. Whether or not one holds that the sole ultimate aim of social policy is to boost individual welfare fairly, there is little to be said for the claim that health is valuable only insofar as it is part of or a cause of welfare or that the burden of disease should be quantified by the consequences of disease for well-being in the circumstances.

Moreover, there are strong reasons not to measure the burden of disease by its consequences for well-being. Broome's measure of health conflicts with most people's judgments concerning the severity of diseases and health states, and it would imply policies that most people would reject. Consider a disability such as paraplegia, to which many people successfully adapt. As people adapt, their well-being improves, but their physical health remains much the same. If one measures the severity of a health state by its consequences for an individual's well-being, then one either has to deny that paraplegia is a significant disability for those who successfully adapt, or one has to maintain, falsely, that people who have such disabilities cannot live excellent lives. If one rejects the second alternative, as one must, then health policy devoted to preventing or curing paraplegia has to be defended mainly in terms of the well-being costs of adapting to inability to walk, the costs to others of accommodations for the disabled, or the diminished contribution those with these disabilities may make to the well-being of others. Rather than jumping through these hoops, one should recognize that paraplegia is a significant disability regardless of the extent to which it may diminish well-being.

Broome does not maintain that his measure of the burden of disease matches our intuitive judgments concerning the severity of diseases and health states, and he could maintain that popular opinion about the significance of common disabilities is mistaken. If he had a well-supported theory and our pretheoretic views about the significance of disease had no rationale, then the conflict of Broome’s views with popular judgment would not be a very serious criticism. But, on the one hand, the common view that health states such as blindness, deafness, and paraplegia are significant disabilities can be explained and rationalized by pointing to the effects of health on things other than well-being, such as opportunity, and, on the other hand, as I argued above, Broome's view is not well-supported.

The conflict between Broome's measure of the burden of disease and people’s judgments concerning health is stark. If one were to measure the burden of disease in the way that Broome defends, two countries that have exactly the same distribution of health states and whose health distribution is changed in exactly the same way by a disease would nevertheless be burdened differently by the disease, whenever its effects on well-being were not the same in both countries. By assumption, if the health states are the same, the health effects of the disease are the same, yet the burden of disease is not the same. For those who want to measure the consequences for health, this result is intolerable. On Broome’s view, in contrast, this result is unsurprising. Broome is, after all, denying that the burden of disease should be calculated in terms of the consequences for population health. So what if the effects of disease on health are just the same? The burden of disease as Broome understands it – that is, the consequences of disease for well-being – is not the same.

It is hard to accept this view of the burden of disease, because people are concerned specifically about health. Learning that the burden of disease conceived of in Broome’s way is greater in one country than another would tell us that the effects of the disease are worse, but it would not tell us that there was a greater health problem or that more resources that are earmarked to address health problems should go to one country rather than the other. A measure of the burden of disease in terms of the consequences for well-being may fail to tell those who are concerned about health what they want to know.

There are also practical problems with Broome’s proposal to measure the burden of disease by the effects of disease on well-being. Though it is hard to assign a scalar measure to health and hence to measure the health effects of disease, it seems to be harder still to assign a scalar measure to well-being. What constitutes a person’s good is at least as multidimensional as what constitutes their health. There are methods of quantifying *preferences*, but preference satisfaction does not coincide with welfare. Furthermore, whereas there are clear constraints on interpersonal health comparisons, since people’s health is the same when they are in the same health state, it is far from clear how to make interpersonal comparisons of preferences or well-being. If it is not possible to measure well-being on at least an interval scale and, in addition, to make interpersonal comparisons of well-being, then it is not possible to aggregate individual well-being or to compare changes in well-being across populations. So it would be impossible to estimate the effect of disease on overall well-being. Even if there were good reason to implement Broome’s proposal, it would be very difficult to do so.

Moreover, possessing an interpersonally comparable interval-scale measure of overall well-being would not by itself get one far, because health economists are in no position to estimate the consequences of diseases, injuries, and risk factors or of health policies on overall well-being. To estimate consequences of a disease or a policy on well-being requires that one compare what well-being would be in the future with or without the disease or the policy. To make such a comparison demands much more than knowledge of the direct effects of diseases or health policies on health states. In addition, one needs estimates of economic growth, technological progress, climate change, educational achievement, political stability, and so forth. One cannot expect health economists to have this knowledge. Though one might reasonably expect them to be able to estimate the effects of diseases or policies on the distribution of health states, one cannot reasonably expect them to be able to estimate the effects of diseases or policies on well-being.

Insofar as policymakers aim to enhance well-being, they need to be able to estimate the well-being consequences of social policies. What they can reasonably expect from health economists are data from which they (the policymakers) can draw inferences concerning what the consequences for well-being will be of diseases or health policies when they are coupled with education policies, agricultural policies, transportation policies, and so forth. They cannot expect health economists to tell them what the consequences for well-being would be.

Instead of defining the mission of the various state sectors (health, education, occupational health and safety, environmental policy, and so forth) to be to promote well-being by manipulating the particular causal factors within the purview of the particular state agency – which seems to be Broome's view ([2], p. 98) – contemporary governments assign different goals to different sectors. They do this because there is no feasible alternative, even if everyone accepted Broome's view that the ultimate goals of policy are well-being and fairness. Of course current practices result in many misdirected efforts and mistaken policies. Coordination among various agencies is needed. But the department of health cannot be feasibly charged with the promotion of well-being by influencing health, while the department of education aims to promote welfare by influencing education, and the department of transportation aims to promote welfare by building roads, transit systems, and airports. Those working in specific departments do not know how to enhance overall well-being, and their clumsy efforts to do so would inevitably collide. The goals of those concerned with health policy are narrower, and the information they need concerns more immediate consequences of policy or disease.

Although these considerations cast doubt on Broome’s claim that the burden of disease should be identified with the consequences of disease for well-being, they leave his critique of health measures intact. Indeed, if anything, they magnify the difficulties. The difficulties facing those who attempt to measure health do not depend on Broome’s assumption that the value of health consists in its bearing on well-being. To measure health and hence to provide a scalar measure of the consequences of disease for health requires that one assign values to kinds of health states, as defined by some health state classification system. But the consequences of tokens of the same health state type and hence the values of the tokens differ depending on the context. Health states types do not have values. How then is it possible to measure health?

This appears to be a very serious problem. How can a health economist assign a single number to many different values? As difficult as it may be to provide a scalar measure of the consequences of disease for well-being in a specific context, it is harder still to provide an overall measure of the various intrinsic and instrumental values of health states across a whole range of contexts.

Although members of the GBD team have not stated the problem as baldly and explicitly as I have, they have proposed two possible solutions. One possibility is to take the value of a health state of a particular type to be the average over all the different contexts of the values of its tokens, where this average is weighted by the frequency of the contexts ([19], p. 34). More formally, let *V*(*h*) be the value (the quality adjustment) of a health state of type *h* and let *v*_*i*_(*h*) be the value of tokens of *h* that occur in context *i*. *v*_*i*_(*h*) would depend on the intrinsic value of *h* in the *i*’th context and on the value of the consequences of *h* in that context, which could include the consequences of *h* for well-being. One could then specify *V*(*h*) = ∑*f*_*i*_*v*_*i*_(*h*) where *f*_i_ is the frequency of the *i*’th context. There is, however, no feasible way of eliciting all the many context-dependent values of health states and then averaging them. Average health state values might instead be inferred from preferences among health states – though such an inference would be precarious and would place heavy demands on the methods employed to elicit those preferences. Although the global weighted average of the values of the tokens will often differ from both the value of any specific token and from the average value health states have in individual countries or regions, relying on these global average values is arguably a reasonable way to quantify the global health effects of diseases, injuries, and risk factors.

The second way of coping with the problem that tokens of the same health state have differing values is to take the value of a health state to depend upon the value of its consequences in some specified “standard” context ([12], p. 304). Provided that the choice of a standard context can be justified, one can also use the standard values of health states to assign a value to the effects of diseases, injuries, and risk factors on the distribution of health states. Whether average or standardized values of health states will serve the other purposes that a summary measure of population health was designed to serve is another question, which I have addressed in another essay ([15]). But if the distribution of contexts is held constant (as it must be in order to implement Broome’s own proposal that one calculate the effects of diseases on well-being), then the effects of diseases on the value of population health can be calculated from their effects on the distribution of health states and the average or standard values of health states.

## Conclusions

Many problems remain, and different measures may be needed for different purposes. Yet the prospects for assessing the burden of disease in terms of the consequences of disease, injuries, and risk factors for health itself are not so bleak as they may have appeared earlier in this essay. Evaluating health states by the weighted average of the values of their tokens or by the value they have in a standard context is a compromise that grows out of the impossibility of quantifying health itself. But it is a compromise worth making, because (in contrast to Broome’s proposal) some measure of health itself is needed for policy purposes.

## Competing interests

There are no conflicts of interest or competing interests.

## Author’s contribution

The author is a philosopher who has worked on issues at the boundaries between economics and philosophy. He is sympathetic to the project of attempting to generate summary measures of population health but also concerned about the conceptual difficulties the project involves.
